# Recent advances in innovative biomaterials for promoting bladder regeneration: processing and functionalization

**DOI:** 10.3389/fbioe.2024.1528658

**Published:** 2025-01-06

**Authors:** Yi Zhang, Fu’an Ding, Junjie Han, Zongliang Wang, Wenjie Tian

**Affiliations:** ^1^ The Second Hospital of Jilin University, Changchun, China; ^2^ Key Laboratory of Polymer Ecomaterials, Changchun Institute of Applied Chemistry, Chinese Academy of Sciences, Changchun, China

**Keywords:** biomaterials, nanomaterials, bladder regeneration, tissue engineering, stem cells

## Abstract

The bladder is a dynamic organ located in the lower urinary tract, responsible for complex and important physiological activities in the human body, including collecting and storing urine. Severe diseases or bladder injuries often lead to tissue destruction and loss of normal function, requiring surgical intervention and reconstruction. The rapid development of innovative biomaterials has brought revolutionary opportunities for modern urology to overcome the limitations of tissue transplantation. This article first summarized the latest research progress in the processing approaches and functionalization of acellular matrix, hydrogels, nanomaterials, and porous scaffolds in repairing and reconstructing the physiological structure and dynamic function of damaged bladder. Then, we discussed emerging strategies for bladder regeneration and functional recovery, such as cell therapy, organoids, etc. Finally, we outlined the important issues and future development prospects of biomaterials in bladder regeneration to inspire future research directions. By reviewing these innovative biomaterials and technologies, we hope to provide appropriate insights to achieve the ultimate goal of designing and manufacturing artificial bladder substitutes with ideal performance in all aspects.

## 1 Introduction

The bladder is an important organ in the human body that stores urine and urinates through contraction (Dyrskjot et al., 2023). Some serious diseases or injuries usually require surgical reconstruction of the bladder, such as congenital malformations, systemic diseases, tumor resection, and accidental trauma (Li et al., 2024). In clinical practice, bladder injury, especially mechanical injury, is more common than ureteral injury or other reasons. According to statistics, urinary tract injuries account for approximately 0.3%–0.8% of all gynecological surgeries, while bladder injuries may also account for 0.05%–0.66% (Zelivianskaia et al., 2022). The source of transplanted tissue for bladder repair remains one of the important challenges that urologists must face. The ideal implants for bladder reconstruction should be easily accessible and suitable for the urinary system environment. Currently, gastrointestinal or mucosal grafts, as heterologous, acellular, biocompatible, biodegradable, and collagen based scaffolds, possess special biochemical components, excellent mechanical properties, appropriate structure and microenvironment. They are suitable for cell adhesion, proliferation, differentiation, and ultimately bladder regeneration when implanted in bladder injury sites. So they are commonly used for the treatment and reconstruction of bladder injuries (Chan et al., 2021).

The structure of the bladder is mainly composed of mucosal layer, muscular layer, and serosal layer (Table 1). Approximately 60%–70% of the bladder wall is composed of muscle tissue, including the inner, middle, and outer layers (Franco, 2023). The outer and inner layers are composed of longitudinal muscle cells, while the cells in the middle layer are circumferential (Sam et al., 2024). The main part of the muscle layer is composed of a bundle of interconnected muscle cells surrounded by collagen. It can elongate and relax over a wide interval length to maintain bladder function during urination and filling (Zhang T. R. et al., 2024). The maximum expansion of the bladder can reach 15 times its original volume, ensuring that it can comfortably accommodate approximately 300–400 mL of urine and ultimately effectively and controllably excrete urine (Weledji et al., 2019). However, various complications related to bladder diseases and injuries can affect the treatment effectiveness and quality of life of patients to varying degrees, including urinary tract infections, metabolic disorders, bladder stones, etc. (Thakare et al., 2021).

**TABLE 1:** Summary of the different layers of the bladder tissue and the factors successfully regenerating a bladder tissue.

Bladder layers	Components	Regenerating factors	References
Mucosal layer	Urinary tract epithelial cells	Bladder progenitor cells, multipotent stem cells, ASCs; decellularized ECM, autologous Biosheet, hydrogels, scaffolds; growth factors, such as bFGF, VEGF, EGFetc.	Sharma et al. (2022), Iimori et al. (2020), Wang et al. (2022c), Kim et al. (2020b), Chen et al. (2022), Suda et al. (2023)
Muscular layer	Inner layer: longitudinal muscle cells	Smooth muscle progenitor cells, stem cells, ASCs, hMDSCs, hMSCs, etc.,; decellularized ECM, patches, scaffolds, hydrogels, nanofibers, etc.,; growth factors, such as bFGF, VEGF, EGF, etc.,; physical stimuli, such as DLSW, infrared therapyetc.	Pokrywczynska et al. (2022), Jin et al. (2020), Zhang et al. (2021), Iimori et al. (2020), Xiao et al. (2021), Wang et al. (2022c), Kim et al. (2020b), Kim et al. (2021a), Feng et al. (2019), Shrestha et al. (2019)
	Middle layer: circumferential muscle cells		
	Outer layers: longitudinal muscle cells		
Serosal layer	Fibroblasts	Bladder progenitor cells, multipotent stem cells, ASCs; decellularized ECM, autologous Biosheet, hydrogels, scaffolds; growth factors, such as bFGF, VEGF, EGFetc.	Sharma et al. (2022), Wang et al. (2022c), Kim et al. (2020b), Chen et al. (2022), Suda et al. (2023)

Abbreviations. ECM, extracellular matrix; ASCs, adipose stem cells; bFGF, basic fibroblast growth factor; VEGF, vascular endothelial growth factor; EGF, epidermal growth factor; hMDSCs, human muscle-derived stem cells; hMSCs, human mesenchymal stem cells; DLSW, defocused low-energy shock wave.

Since the 1980s, tissue engineering techniques based on scaffold materials, cells, and bioactive growth factors have brought new opportunities for bladder regeneration. In particular, decellularized natural extracellular matrix (ECM) and biodegradable polymers can serve as three-dimensional (3D) porous scaffolds, initially providing stable spatial support for damaged bladder tissue, and then stimulating and supporting tissue regeneration through the synergistic effect of cells and bioactive factors, mediating cell-matrix interactions, guiding tissue formation to replace or reconstruct bladder wall structure and function (Ajalloueian et al., 2018; Casarin et al., 2022; Sharma and Basu, 2022). Among them, acellular ECM, hydrogels, nanomaterials and porous scaffolds were developed for bladder regeneration to provide spatial support and functional guidance, and achieved good results. In addition, some emerging strategies have also been developed or combined with biomaterials for bladder regeneration and function recovery, such as cell therapy, organoids, exosomes, etc. (Garioni et al., 2023; Minoli et al., 2023; Isali et al., 2024). Therefore, in this review, we will focus on summarizing the research progress of innovative biomaterials in the past 5 years or so, especially in terms of processing approaches and functionalization, to identify urgent challenges that need to be overcome and addressed, and to anticipate their potential application prospects in bladder regeneration and functional recovery.

## 2 Acellular ECM

Among numerous biomaterials, acellular ECM holds a leading position in bladder regeneration due to its presence of various intrinsic bioactive factors (Yang T. et al., 2023). It can be obtained by appropriate decellularization treatment of various tissues, such as small intestinal submucosa (SIS), bladder, colon, prepuce, skin, etc. (Hu et al., 2022; Wang X. et al., 2022; Golebiowska et al., 2024). Among them, using suitable acellular ECM biomaterials to regenerate bladder wall muscles has become a very feasible choice for urologists to rebuild bladder walls or enhance bladder muscle bundles in bladder diseases or injuries. It can not only be in the form of micro/nano scaffolds or patches, but also in the form of hydrogel, and contains rich natural ECM intrinsic active ingredients (Zhang et al., 2022). In this section, we will mainly discuss the forms of scaffolds and patches for bladder regeneration. Later, we will introduce the application form of hydrogels.

### 2.1 Acellular matrix derived from luminal tissues

**FIGURE 1:**
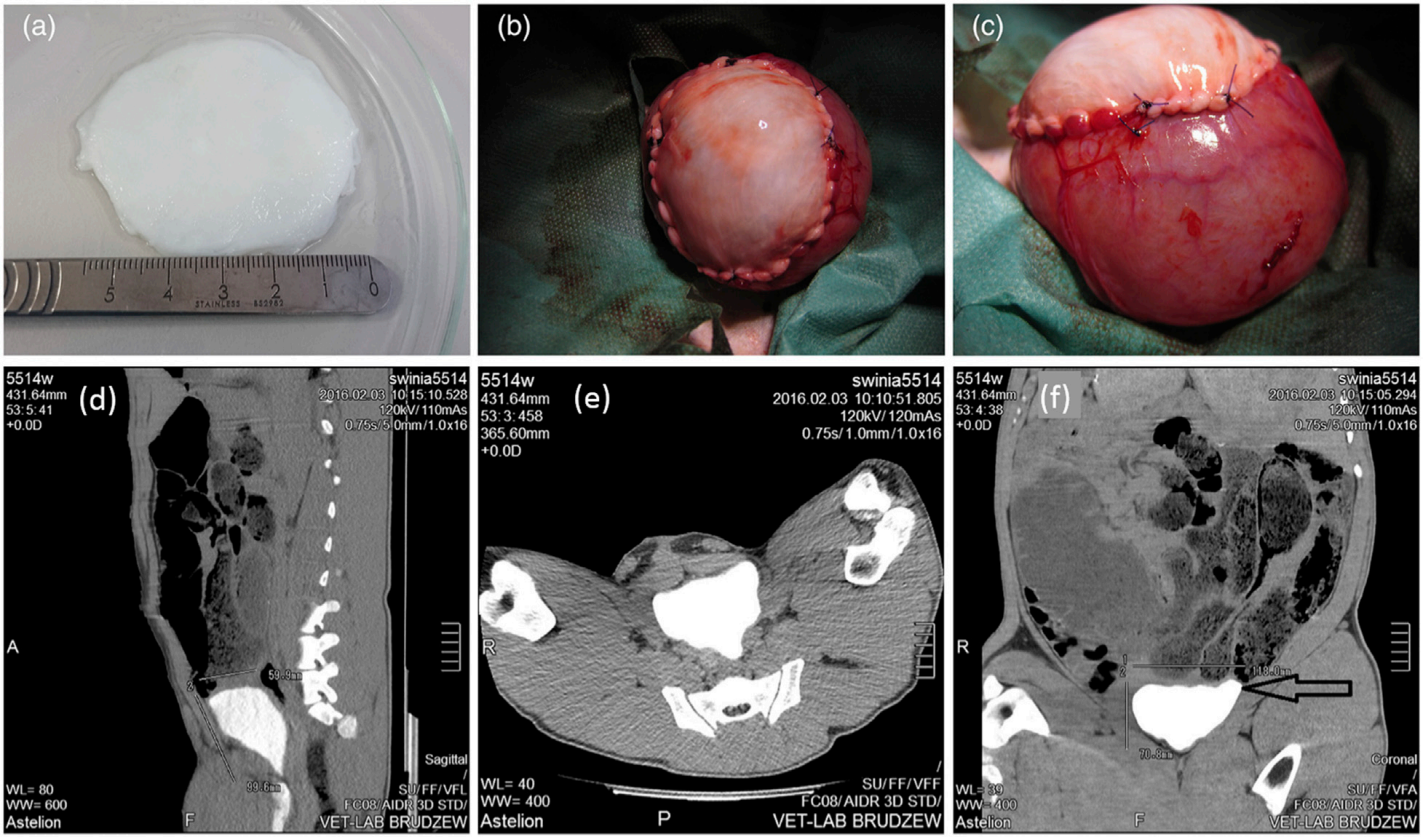
General observation of bladder acellular matrix (BAM) prepared for bladder reconstruction **(A)**. Implantation of BAM into the site of bladder resection **(B)**. The BAM graft was sutured with non absorbable sutures to the edge of the bladder resection **(C)**. **(D–F)** Computed tomography images for 6-month follow-up after BAM implantation surgery. Adapted from (Pokrywczynska et al., 2022), Copyright 2021 John Wiley and Sons.

As a representative, bladder acellular matrix (BAM) is the most commonly used tissue source material. For example, Pokrywczynska et al. evaluated the effect of BAM on the reconstruction of clinically significant large bladder wall defects in pig models through a 6-month *in vivo* observation (Figure 1) (Pokrywczynska et al., 2022). Researchers obtained bladder samples from 10 pig donors and used chemical methods to remove all cellular components from the bladder tissue to prepare a biological BAM scaffold. Then, the obtained white translucent appearance and thickness of about 3–5 mm BAM scaffolds were implanted into 10 pigs that underwent partial cystectomy (Figures 1A–C). Six animals survived until the end of the 6-month reconstruction observation period. Four other pigs died during the observation period due to reasons such as cracked anastomosis or blocked ducts. The function of the regenerated bladder of the successfully surviving pigs was normal, and the CT image of the upper urinary tract showed no signs of dilatation of the renal pelvic hiatus system or urinary retention (Figures 1D–F). The urethral epithelium completely covered the luminal surface of the BAM grafts. Smooth muscle regeneration and bladder wall reconstruction were also clearly visible. This indicates that BAM is a promising biomaterial for reconstructing large bladder wall defects, and it can also be further used to carry and deliver cells to enhance bladder regeneration. In addition to the source of pig bladder tissue, researchers have also developed decellularized goat bladder scaffold, which not only retained the tissue structure and ECM composition of the bladder after removing cellular components, but also had appropriate mechanical properties (Vishwakarma et al., 2020). Subsequently, human umbilical cord blood mesenchymal stem cells (UCMSCs) were seeded onto the decellularized bladder scaffold, achieving satisfactory cell arrangement and proliferation. This biologically manufactured humanized bladder construct provides a feasible allogeneic graft for enhancing future bladder regeneration applications. To improve the biological function of BAM biomaterials, another study incorporated lipid nanospheres containing myogenic differentiation one activated RNA (NPMyoD) into BAM graft and inoculated them with adipose stem cells (ASCs), successfully achieving muscle regeneration, bladder morphology repair, and urinary function in a rat bladder defect model (Jin et al., 2020). Overall, the detailed analysis of the preclinical, morphological, and molecular biological aspects of BAM in bladder reconstruction clearly demonstrates the enormous potential of BAM materials in clinical practice. At present, the urgent issue facing clinical translation of this technology is how to prevent the occurrence of fibrosis in the transplant area. With the external stimulation of implants in the body, a series of complex biological reactions may occur in the surrounding tissues, triggering a series of repair reactions. If the reaction is excessive or imbalanced, it may lead to excessive proliferation of fibrous tissue, resulting in fibrosis. Fibrosis not only affects the stability and function of implants, but may also lead to a series of complications such as pain, thereby affecting the patient’s postoperative experience. Perhaps appropriate cell culture before BAM implantation can solve this problem. Moreover, further strengthening of graft revascularization is needed to reduce ischemic necrosis and fibrosis of the graft *in vivo* (Willacy et al., 2024).

Researchers also prepared another type of natural tissue colon by perfusion decellularization and implanted it into the site of partial cystectomy (Kajbafzadeh et al., 2016). After 1 month of implantation, new cells were observed to grow into the implants. At 3 months, the decellularized colon produced a continuous transitional epithelium structure similar to that of a natural bladder. The bladder was normally spherical in shape, but no stone formation, necrosis, or implant rejection was observed. It also regenerated urinary tract epithelium with a morphology very similar to natural tissue, reconstructed blood vessels, and infiltrated smooth muscle cells into the implants. The decellularized colon matrix, as a natural source, provides a feasible candidate for the treatment of bladder diseases and paves the way for future applications. This also indicates that it has been proven to be a biocompatible biomaterial comparable to BAM, capable of reproducing large bladder wall defects with clinical symptoms (Pokrywczynska et al., 2022).

Lyoplant (B. Braun, Germany) is a biocompatible collagen mesh derived from bovine pericardium, which has been used to reconstruct full-thickness maxillary defects and congenital abdominal wall defects (Meyer et al., 2006; Meyer et al., 2010; Riva et al., 2022). In a rat experiment, Winde et al. implanted Lyoplant collagen mesh into the bladder defect site and conducted histological and immunohistochemical examinations 6 weeks later (Winde et al., 2019). It was found that all rats exhibited physiological growth and behavior after surgery, and no wound healing complications, wound infection, or hernias were observed. Implants exhibited sufficient tissue binding in all cases. Immunohistochemical analysis further confirmed significant cell infiltration and neovascularization. Their findings suggest that the collagen-based Lyoplant appears to be a potential candidate for bladder regeneration. In terms of functional modification of decellularized matrix, natural biological crosslinking agent procyanidins have been used to crosslink SIS patches, endowing them with anti-calcification, anti-inflammatory, and antioxidant properties, improving bladder repair efficiency, promoting smooth muscle regeneration, and restoring bladder function in rabbit models (Zhang et al., 2021).

### 2.2 Acellular matrix derived from skin tissues

In addition to acellular matrix derived from various luminal tissues, multiple studies have also prepared decellularized matrix from different parts of skin tissues. For example, a scaffold based on prepuce 3D collagen was prepared and inoculated with ASCs, which were then applied for bladder wall regeneration (Kajbafzadeh et al., 2014). The scaffold was produced by decellularizing the prepuce of children, which was obtained under circumcision. The collage fibers of the scaffold had good organization and orientation that was very similar to natural ECM. After implanting the ASC-seeded scaffold between the bladder mucosa and the seromuscular layer, the formation of muscle bundles was clearly observed. This might be due to the differentiation of ASCs into mature smooth muscle cells (SMCs) *in vivo*, leading to reduced fibrosis and ultimately resulting in bladder wall regeneration. However, due to insufficient research depth and limitations in donor material sources, prepuce scaffolds have not yet been commercialized, and there is an urgent needed to translate this technique into clinic practice.

Collagen membrane scaffolds derived from bovine skin have also been prepared and used as targeted delivery vehicles for growth factors to promote bladder regeneration (Chen et al., 2010). In this targeted regeneration system, the researchers utilized collagen binding domains fused to the N-terminus of natural bFGF (CBD-bFGF) to specifically bind to collagen scaffolds, improving loading efficiency and prolonging release time. After implantation of partially excised rat bladder, the collagen scaffold loaded with CBD-bFGF regenerated bladder tissue similar to natural tissue structure, producing more blood vessels and inwardly growing smooth muscle cells. The urodynamic test results indicated that the regenerated bladder had good adaptability, larger capacity, and better compliance. This indicates that this targeted regeneration system can better induce bladder regeneration at the site of injury.

**FIGURE 2:**
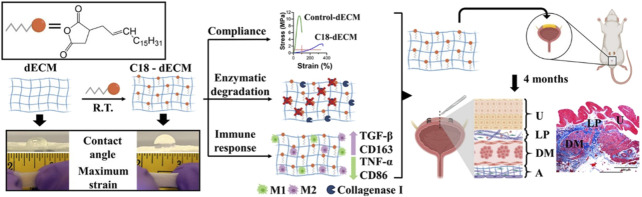
Schematic illustration of the preparation of highly stretchable decellularized extracellular matrixes (dECMs), which were modified with long aliphatic chains for bladder regeneration. Adapted from (Sharma et al., 2022), Copyright 2022 American Chemical Society.

Another study compared the decellularized matrix of different types of luminal tissues and skin tissues from different species. In this study, as shown in Figure 2, researchers selected five commercially available decellularized ECM (dECMs) for clinical use, including decellularized porcine SIS (dSIS), decellularized urinary bladder matrix (dUBM), decellularized bovine pericardium (dBP), decellularized bovine dermis (dBD), and decellularized human dermis (dHD). Then, these dECMs were modified with long aliphatic chains (C9, C14, and C18), which served as transient crosslinkers by chemically crosslinking with the amino groups in dECMs (Sharma et al., 2022). After modification, dECMs showed significant resistance to enzymatic degradation of collagenase type I and extended its lifespan. Compared with the control group, the hydrophobicity, stretchability, and compliance were all improved at lower strain values below 10%. In addition, the modified dECMs exhibited better elongation of over 200% and viscoelastic dissipation in physiological microenvironments. Among them, the C18 modified dECMs showed the best compliance, with a stress of 0.28 MPa at 100% elongation, equivalent to the performance of rabbit bladder tissue (0.25 MPa at 100% elongation). The hydrophobicity of the modified dECMs could alleviate the pro-inflammatory response of macrophages, and the implants did not exhibit *in vivo* rejections such as excessive collagen deposition or the formation of foreign giant cells. Further morphological analysis of the implanted rat bladder confirmed that the presence of aliphatic molecules did not have adverse effects on the regeneration process of the urinary tract epithelium and bladder wall, but rather had a supportive effect. Overall, the modification of decellularized matrix by long-chain aliphatic molecules significantly improved support for urine storage and enhanced the compliance and mechanical properties of the regenerated bladder, enabling it to better withstand cyclic loading and unloading, supporting cell-matrix interactions in the regenerated bladder tissue, regulating the host immune system, and creating an anti-fibrotic regeneration microenvironment. Therefore, it is of great significance for the engineering of high-capacity load-bearing deformable tissues such as the bladder.

### 2.3 Cell-free tissue engineering technology

Unlike decellularized matrix based on various tissues, *in vivo* tissue structure technology based on cell-free tissue engineering can produce collagenous tissues by subcutaneous implantation of specific molds. This technology, known as “in-body tissue architecture (iBTA) technology”, has been developed by subcutaneously implanting well-designed molds into animal bodies to form autologous collagenous tissues (Takiyama et al., 2016). This process does not involve complex decellularization procedures, and the main components of the formed collagen tissue are fibroblasts and ECM rich in type I collagen. Its burst strength can exceed 200 mm of mercury, and its size and thickness can be adjusted by changing the molds (Terazawa et al., 2020). In a study, Akiyoshi and colleagues prepared a canine Biosheet implant by subcutaneously embedding a mold into beagle dogs and removing it after 8 weeks. Subsequently, they implanted the autologous Biosheet into partially excised canine bladder wall of the same size for repair (Iimori et al., 2020). After the surgery, no rupture of the implanted Biosheet or leakage of urine into the peritoneal cavity was observed. The authors also did not find any chronic inflammation or rejection reactions. After 4 weeks of implantation, the urinary tract epithelial cells formed a multicellular layer, completely covering the surface of the implanted Biosheet. After 12 weeks, muscle cells and newly formed microvessels were clearly observed in the implantation area. The Biosheet implant prepared in this study demonstrates excellent biocompatibility as a bladder reconstruction scaffold, indicating its suitability for full thickness bladder wall replacement.

Overall, decellularized ECM biomaterials do have unparalleled innate advantages in bladder regeneration, such as retaining the elastic fibers of natural ECM to provide sufficient mechanical strength, retaining the 3D structure and functional proteins of ECM (collagen, elastin, glucosamine, etc.) to support cell growth and differentiation, reducing transplant rejection due to the low immunogenicity of removing cellular components, and being easy to prepare and store for clinical applications. However, decellularized ECM biomaterials do have some drawbacks, including complex preparation techniques such as tissue-specific decellularization treatment and directional freezing of porous materials, as well as high production cost due to the specificity of technology and materials. However, in terms of the material itself, decellularized ECM still has enormous potential in multiple fields. It is believed that its practicality will be further validated through clinical applications and research development stages.

## 3 Hydrogels

As an attractive soft material, hydrogel has been widely explored and applied in the biomedical field in recent years due to its unique characteristics such as high water content, flexibility, biocompatibility, etc. (Wu et al., 2024). Hydrogel is a kind of gel with 3D network structure and strong hydrophilicity. Its crosslinking network makes the hydrogel swell and retain a large amount of water, which makes it very similar to the characteristics of human soft tissue (such as bladder), so it is considered to be an ideal material for soft tissue repair (Shen et al., 2022; Mehta et al., 2023). Although many hydrogel materials have received extensive attention in the field of tissue repair due to their outstanding characteristics, in terms of bladder tissue regeneration, whether they are derived from acellular matrix hydrogels or natural and synthetic polymer hydrogels, they usually need to be used in combination with other biomaterials or deliver bioactive substances to achieve better repair effects (Fu et al., 2023; Oh et al., 2023; Liu et al., 2024). In this section, we will mainly discuss two types of hydrogel biomaterials: acellular matrix hydrogels and natural or synthetic polymer hydrogels. However, the combination of different hydrogel materials is inevitable.

### 3.1 Acellular matrix hydrogels

As a natural ECM mentioned above, BAM hydrogel is widely used as a scaffold to promote bladder tissue reconstruction and repair because of its intrinsic bioactive components (Yang T. et al., 2023). In one study, Liu et al. prepared BAM hydrogel with customized porous structure by adjusting the concentration of BAM, overcoming the limitations of its dense structure, and enhancing the angiogenesis potential of BAM (Liu et al., 2019). They found that 6 mg/mL BAM significantly improved the mechanical strength and gel speed of BAM hydrogel, and its pore size decreased with the increase of concentration, which was better than the commercially available collagen hydrogel (2.5 mg/mL). When porcine iliac endothelial cells (PIECs) were loaded, the BAM hydrogel led to better cell growth and showed a higher density of live spindle PIECs, with an average length of about 50 μm. While the PIECs in the collagen hydrogel of the control group was spherical, with a length of about 30 μm. In additional, compared with PIECs/collagen and BAM hydrogel without cells, PIECs/BAM hydrogel had a higher rate of revascularization. A series of comparative analysis showed that the higher angiogenesis potential of the PIECs/BAM hydrogel might be due to the increased proliferation of PIECs and the promotion of angiogenesis by the residual growth factors in the BAM hydrogel.

In addition to using BAM hydrogel alone to support cell delivery, it was also used in combination with other natural polymer hydrogels. For example, a hybrid composite scaffold composed of BAM, alginate dialdehyde and gelatin gel, as well as silk mesh was prepared for bladder regeneration (Xiao et al., 2017). Adipose derived stem cells (ASCs) were encapsulated in the scaffold, forming a tri-layered scaffold that appropriately degraded, reduced fibrosis and inflammation levels, and promoted bladder morphology and histological repair by enhancing smooth muscle regeneration, angiogenesis, and nerve innervation. This might be due to the superior mechanical properties of this sandwich-like scaffold, where cell encapsulation enhanced the expression of vascular endothelial growth factor (VEGF) mediated by the SDF-1α/CXCR4 pathway, thereby promoting angiogenesis and bladder regeneration.

In order to further enhance the biological activity and specific functions of hybrid hydrogels, they are also used to deliver bioactive substances to promote the repair of tissue injury, such as exosomes (Fan et al., 2024; Han et al., 2024). Exosomes are nanoparticles with a particle size of approximately 50–150 nm, originating from different cellular sources traditionally considered as medical waste. They typically contain various bioactive molecules such as peptides, proteins, nucleic acids, lipids, etc., which can mediate intercellular communication and further act as receptor cells to exert paracrine or endocrine effects (Kalluri and LeBleu, 2020). The signaling role of MSC-derived exosomes in the development, growth, and maturation of tissue neovascularization networks is extremely important, and they have been widely used in regenerative medicine and tissue engineering, regulating angiogenesis, or as drug delivery carriers (Rezaie et al., 2022). For example, in a study, Xiao et al. encapsulated exosomes from ASCs in a three-layer hydrogel scaffold composed of BAM, gelatin and alginate to give full play to the characteristics of exosomes and enhance the biological function of the hydrogels (Xiao et al., 2021). The composite scaffold significantly enhanced the proliferation, migration and tubular structure formation of human umbilical vein endothelial cells (HUVECs), thereby promoting angiogenesis, improving the reconstruction and functional recovery of bladder epithelium, smooth muscle and nerve fiber structures, and alleviating fibrosis and inflammation. The potential molecular mechanism might be that miRNA-126 contained in exosomes inhibited human G-protein signaling 16 (RGS16) and activated the CXC chemokine receptor/stromal cell derived factor-1α (CXCR4/SDF-1α) pathway, further upregulating VEGF secretion by the phosphorylation of extracellular signal-regulated kinase 1/2 (ERK1/2).

### 3.2 Natural or synthetic polymer hydrogels

Natural or synthetic polymer hydrogels have also been used to simulate the structure of natural ECM and regulate cell growth and tissue repair. For example, in a work, a multilayer biomimetic scaffold composed of oxidized dextran/carboxymethyl chitosan hydrogel and poly(ε-caprolactone) (PCL) nanofibers was prepared for bladder regeneration (Zhao et al., 2024). In this material system, bone marrow homing peptide (BMHP) and vascular endothelial growth factor mimic peptide (VP) were respectively incorporated into the hydrogel to recruit endogenous stem cells and promote angiogenesis. The composite scaffold not only had good mechanical properties, but also had a longer retention time of peptides. The loading of peptides accelerated bladder regeneration, enhanced the contractility of smooth muscle cells, inhibited fibrosis, and improved nerve innervation function. Overall, this novel functional scaffold has the potential for tissue reconstruction in the urinary system. In another study, Jiang et al. prepared a composite hydrogel based on synthetic polymer materials. They first obtained poly(lactic-co-glycolic acid) nanoparticles (PLGANPs) containing VEGF or VEGF and basic fibroblast growth factor (bFGF). The PLGANPs loaded with VEGF and/or bFGF were suspended in Pluronic F127 solution and quickly embedded into an acellular bladder tissue to construct a thermosensitive gel system for promoting bladder tissue regeneration (Jiang et al., 2015; Jiang et al., 2016). After 12 weeks of implantation in the rabbit bladder, the functional composite scaffold continuously released growth factors, increased microvascular density, effectively regenerated the cell layer of urinary tract epithelial cells and smooth muscle cells, reduced graft contracture, and promoted bladder tissue reconstruction. Especially, the scaffold that simultaneously co-delivered these two factors exhibited programmed release and had a synergistic effect on bladder regeneration (Jiang et al., 2016). However, when promoting the clinical transformation of this gel system delivering bioactive substances, there are still some problems to be overcome, such as how it affects the long-term recovery of bladder function, the monitoring of multiple tumor markers after implantation, the interaction mechanism between host cells and gel implants, and the improvement of the regeneration process.

Although we have made every effort to review and discuss the above hydrogels for bladder regeneration in the past 5 years or so, there are still few hydrogel products that have really successfully achieved satisfactory clinical repair effects. Of course, the reasons may be complex, including the dynamic and mechanical properties of bladder biological tissue itself. This should take into account the extension of microstructure and appropriate mechanical properties, as well as specific tissue induction ability, so this puts forward higher requirements for tissue repair hydrogels (Ajalloueian et al., 2018). This also urges us to make better use of the good development trend of biomaterials and engineering science to find and develop functional hydrogels that are more suitable for bladder tissue engineering.

## 4 Nanomaterials

Nanomaterials are another kind of biomaterials which are widely used in bladder regeneration except hydrogels and acellular ECM. It can include electrospun nanofibers and inorganic nanomaterials. Electrospun nanofibers are an ideal material system for constructing and regenerating the bladder injuries (Abadi et al., 2022). Ultra fine nanofibers have microstructures with diameters ranging from tens of nanometers to micrometers and extremely high specific surface areas, which are very similar to natural ECM, making them suitable for providing a simulated *in vivo* microenvironment for cell growth (Wan et al., 2022; Yang S. et al., 2023). Inorganic nanomaterials are also used in bladder regeneration or targeted delivery of drugs and growth factors (Cui et al., 2023; Zhang et al., 2023; He et al., 2024). They have unique physical and chemical properties that can promote cell adhesion, proliferation, and differentiation, enhance biocompatibility and improve repair capabilities (Ma and Wu, 2022). They can be combined with other polymer materials to form nanocomposites, thereby personalized repair can be achieved by precisely controlling the size, porosity, and mechanical strength of the composite scaffold.

### 4.1 Electrospun nanofibers

As mentioned earlier, in the process of bladder regeneration, nanofiber scaffolds not only provide micro/nano structural support that simulates natural ECM, but also serve as delivery carriers for bioactive molecules to enhance the biological functions of materials. This is because rebuilding the neovascularization network during bladder regeneration requires the action of bioactive factors to provide sufficient nutrient and growth regulatory signals. Therefore, the delivery of biological factors is crucial, especially VEGF, which is an important regulatory factor for angiogenesis (Li et al., 2023). Its load and release are beneficial for attracting endothelial sprouts to the regeneration area. It can be expected that combining exogenous VEGF with biological scaffolds in an appropriate manner and releasing it in a controlled manner is of great significance for bladder regeneration.

**FIGURE 3:**
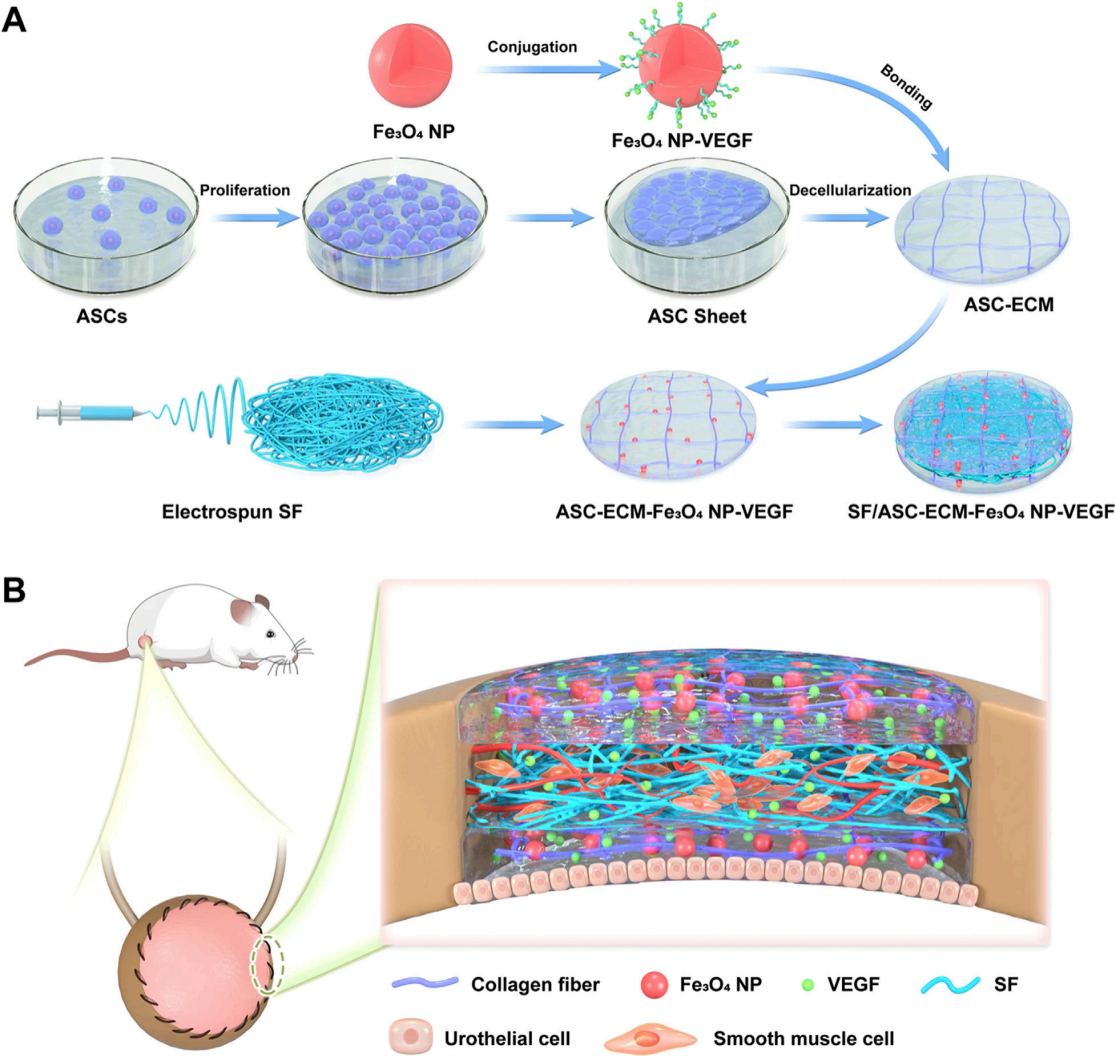
Schematic illustration of the manufacturing and application of a proangiogenic bladder graft based on cell sheet and nanomaterial technology. **(A)** The ECM scaffold was obtained via decellularizing ASC sheets. The Fe_3_O_4_ nanoparticles (NPs) bound with VEGF were covalently conjugated to the ECM scaffold, forming the ECM-Fe_3_O_4_NP-VEGF scaffold. Subsequently, SF nanofibers and ECM-Fe_3_O_4_NP-VEGF scaffold were assembled into SF/ASC-ECM-Fe_3_O_4_-VEGF scaffold. **(B)** The SF/ASC-ECM-Fe_3_O_4_NP-VEGF scaffold was implanted in an SD rat bladder defect model to evaluate its therapeutic effect. Adapted from (Wang Y. et al., 2022), Copyright 2022 Springer Nature.

In one study, superparamagnetic iron oxide nanoparticles (Fe_3_O_4_NP) were incorporated into electrospun silk fibroin (SF) nanofiber scaffold to achieve VEGF loading. The prepared ECM-like bioactive scaffold exhibited excellent mechanical properties, adjustable biodegradability, and good biocompatibility, which can promote angiogenesis in bladder regeneration (Figure 3) (Wang Y. et al., 2022). The authors first obtained an acellular ECM scaffold based on decellularization of ASC sheets, and then chemically conjugate it to VEGF-coupled Fe_3_O_4_NP (Fe_3_O_4_NP-VEGF). In order to further improve the mechanical properties of the biological scaffold, they pressed electrospun SF nanofibers onto both the upper and lower surfaces of the bioactive scaffold to prepare a biomimetic ECM-like scaffold. The bioactive scaffold promoted the adhesion and proliferation of endothelial cells, significantly accelerated *in vivo* angiogenesis in a rat bladder enlargement model, and also promoted the regeneration of urinary tract epithelium and smooth muscle. The results indicated that this biomimetic scaffold had enormous clinical application potential. This study is a typical case of combining polymer nanofibers, decellularized ECM matrix, various cellular active ingredients, and growth factors.

Natural polymer materials are also commonly used in combination with synthetic polymer materials to prepare nanofiber scaffolds for bladder repair. As a representative of numerous natural polymer materials, hyaluronic acid (HA) is a hydrophilic polysaccharide with good biocompatibility, biodegradability, and non toxicity (Miglani et al., 2023). It can support the elasticity of tissue structure and maintain cell vitality. For example, a coaxial biomimetic nanofiber scaffold was prepared by electrospinning, with an outer layer of HA and an inner layer of poly(L-lactide-co-caprolactone) (PLCL), exhibiting high porosity and high surface area to volume ratio (Feng et al., 2019). In a rat bladder enlargement model, the scaffold provided specific physical strength sufficient for early separation of urine and stimulated the formation of bladder smooth muscle layers. Subsequently, in another study, the authors combined electrospun PLCL nanofiber mats with HA colloidal solution to form multi-layer nanofibrous patches, and added adipose tissue extract (ATE) as a bioactive additive (Wang J. et al., 2022). Researchers found that this bioactive patch with a hierarchical microstructure significantly promoted the proliferation, migration, and angiogenesis of endothelial cells (ECs). The various cytokines rich in ATE also provided a stable mechanical microenvironment and important biochemical factors for bladder regeneration, which could promote neovascularization and maintain the pluripotency of stem cells.

In another study, to overcome mechanical and biocompatibility issues and effectively construct biomaterials with appropriate thickness as suitable scaffolds for bladder smooth muscle regeneration, Lee and colleagues prepared multi-layer PCL nanofibers and inoculated them with human muscle-derived stem cells (hMDSCs) (Shrestha et al., 2015). When implanted in a bladder with preserved mucosa, it was found that hMDSCs seeded on the multi-layer PCL exhibited effective cell proliferation and improved smooth muscle cell regeneration by promoting angiogenesis. Similarly, multilayer PLCL nanofiber sheets were prepared and inoculated with ASCs to improve bladder regeneration and function in rats (Shrestha et al., 2019). The differentiation of ASCs into smooth muscle cells significantly helped to restore the contractility and compliance of the damaged bladder. In addition, the combination of polymer nanofibers with decellularized natural bladder matrix has also achieved good results. In this work, the authors utilized the excellent mechanical and biological properties of SF to electrospun well-arranged nanofibers onto a layer of natural bladder decellularized matrix (Li et al., 2015). The neat arrangement of nanofibers in multiple layers with a grid-like structure significantly improved the tensile performance and retention of the composite scaffold, thereby achieving good suturing with tissue during implantation. The scaffold had good biocompatibility and supported the adhesion, proliferation and infiltration of PIECs in the scaffold when loaded with VEGF. This work can be seen as another representative material system that integrates polymer nanofibers, decellularized ECM, and growth factors, once again confirming the important role of the design of functional hybrid biomaterials in bladder tissue engineering and regenerative repair.

### 4.2 Inorganic nanomaterials

In the aforementioned sections, some inorganic nanomaterials applied to bladder tissue engineering have been discussed, including PLGA nanoparticles, lipid nanospheres, and Fe_3_O_4_ nanoparticles. In this section, we will focus on discussing two other types of inorganic nanomaterials.

Among numerous inorganic nanomaterials, graphene is a representative material that, due to its unique nanostructure, can achieve the effect of interacting with single cells and provide sufficient cell contact interfaces (Gao et al., 2024). When the graphene layer filled with cells acted as a nanoscale implant, it can transmit electrical stimulation, rebuild cell communication, and regulate cell behavior, such as contraction (Qian et al., 2021). In a study, biocomposites prepared by combining graphene and amniotic membrane established an interface between cells and external electrical stimulation (Adamowicz et al., 2020). After inoculation with SMCs and porcine urothelial cells (UCs), it was found that the presence of graphene layer significantly increased the conductivity of the biocomposites and enhanced the organized growth pattern of SMCs and UCs on the graphene covered surface. When external electric field stimulation (EFS) was applied, it also led to an increase in SMC growth and linear arrangement, and the pressure changes of SMC contraction were recorded. This indicated that the introduction of graphene significantly improved the interfacial interaction between cells and materials, providing a successful neural network for bladder tissue engineering.

In addition, an acellular nanocomposite scaffold (ANS) was constructed based on zeolite imidazole salt skeleton-8 (ZIF-8) nanoparticles loaded with exosomes from stromal vascular fraction (SVF). Then, the nanoparticles were encapsulated in PLGA microspheres and subsequently incorporated into an acellular bladder matrix (Zhao et al., 2023). This scaffold could slowly degrade and release a large amount of SVF exosomes to promote tissue regeneration. Even after long-term cryopreservation, the acellular nanocomposite scaffold still exhibited biological activity and strong angiogenesis ability in a rat bladder injury model, inducing macrophage M2 polarization to promote tissue regeneration and restore bladder function. This study indicates that the functionality of this nanocomposite scaffold is similar to that of stem cells, while also avoiding the drawbacks of cell therapy.

Although the drawbacks of nanofibers are quite obvious, such as easy breakage and structural damage, only supporting the growth of surface monolayer cells, making it difficult for cells to penetrate into the scaffold, their significant advantages also make these materials play an important role in tissue regeneration (Anjum et al., 2022). Inorganic materials in this field are rarely used alone, but in combination with other matrix materials. The advantages of inorganic nanomaterials inevitably determine their crucial role in bladder regeneration. Due to their biomimetic structure that can replicate the microenvironment of natural tissues, they undoubtedly can significantly improve cell adhesion, proliferation, and differentiation (Mekuye and Abera, 2023). The delivery of biomolecules or stem cells can significantly enhance their repair ability. Of course, the drawbacks of inorganic nanomaterials in tissue regeneration cannot be ignored, such as the issue of biological barriers. That is because nanoparticles are difficult to break through the natural biological barrier formed by the bladder wall mucosal layer, resulting in low bioavailability (Fernandes et al., 2020). Furthermore, the penetration depth of tissues is limited. For example, the tissue penetration ability of near-infrared first zone light is limited, while the penetration depth of near-infrared second zone light is very deep, but the equipment and technical requirements are high, which limits the application of infrared responsive inorganic nanomaterials (Pei et al., 2023; She et al., 2024). However, inorganic nanomaterials still have broad application prospects in bladder regeneration, such as well-designed pH-, redox- or photo-responsive intelligent nanoparticles, which has significant potential implications for bladder tissue regeneration and tumor treatment.

## 5 Porous scaffolds

Porous scaffold are also an essential form of biomaterial for bladder regeneration. They have 3D interconnected spatial structure, appropriate porosity, and mechanical strength to support cell growth, infiltration and tissue regeneration, and are widely used in the engineering various tissues (Peng et al., 2023; Santos-Silva et al., 2024; Zhang M. et al., 2024). Moreover, in recent years, the methods for preparing porous scaffolds have become increasingly diverse, including solvent evaporation, phase separation, 3D printing, and so on.

A research team led by Dr. Arun Sharma has developed a synthetic flexible “bladder patch” that provided better standard surgery for severe bladder dysfunction in long-term large animal models (Bury et al., 2024). The “patch” was a flexible poly(1,8-octamethylene-citrate-co-octanol) (PRS) scaffold prepared by evaporating the solvent in a glass mold at room temperature. And it was seeded with the patient’s own bone marrow stem cells (BMSCs) and then sutured onto the bladder injury site. It helped promote strong long-term functional bladder tissue regeneration, restore function, and facilitate the regeneration of existing tissues. This new platform may provide a better alternative to current surgeries that involve removing a portion of a patient’s intestine and connecting it to the bladder. Compared with the intestinal patch method, the new approach significantly reduces complications during follow-up. This highly transformative long-term research is paradigm shift in this field. It provides a unique and innovative method of bladder tissue regeneration for patients with severe bladder dysfunction, and we are pleased to take the next step in clinical trials for these patients.

To overcome the fibrosis and graft contraction problems caused by traditional scaffold transplantation in bladder repair, researchers prepared PCL scaffold with a gradient structure and modified it with growth factors to enhance bladder tissue regeneration in a rat model (Kim H. Y. et al., 2020). The growth factor modified scaffold used three types of growth factors, namely, bFGF, VEGF, and EGF (epidermal growth factor). The regenerated urinary tract epithelium by the growth factor modified scaffold showed high level of organization, high ECM density, high level of angiogenesis and smooth muscle bundle, as well as high expression transcripts related to smooth muscle and urinary tract epithelial differentiation. This indicates that the gradient structured PCL scaffold functionalized with growth factors can improve bladder regeneration in both functional and histological aspects. Recently, another scaffold with conductivity and strong mechanical properties has also been developed for bladder tissue regeneration (Ameer et al., 2024). In this study, Ameer et al. developed a scaffold composed of a hydrophobic conductive polymer poly (3,4-ethylenedioxythiophene) (PEDOT) and a similar hydrophobic citrate based elastomer poly (octamethylene-cooctanol citrate) (POCO), which were polymerized *in situ* and significantly promoted bladder repair and functional recovery. This is the first report of a biodegradable elastic electroactive scaffold that can reconstruct the anatomical structure and biological function of the bladder without cells.

In this section, we discussed the current research on porous scaffolds and introduced their typical advantages and disadvantages. Its advantages include good biocompatibility, mimicking the highly interconnected structure of natural ECM for cell adhesion, proliferation and migration, providing a favorable environment for tissue regeneration, and excellent mechanical properties for necessary structural support in tissue regeneration, especially in bladder epithelium and muscle tissue regeneration (Gong et al., 2022; Wang et al., 2024). However, regardless, as a promising tissue engineering material, the combination of porous scaffolds with other biomaterials will demonstrate broad prospects in bladder tissue regeneration, with appropriate structural modifications and enhanced biological activity.

## 6 Emerging strategies

### 6.1 Cell therapy

Cell therapy is a treatment method that uses autologous or allogeneic cells to repair tissues and organs. It is widely used in diseases such as tissue and organ damage, bone marrow transplantation, myocardial infarction, and malignant tumors (Kimbrel and Lanza, 2020; Bashor et al., 2022; Hoang et al., 2022). Among them, using stem cells for cell therapy is a promising therapeutic strategy for tissue regeneration and disease treatment (Bydon et al., 2024; Pan et al., 2024).

For instance, Kim et al. found that the transplantation of multipotent stem cells (M-MSCs) derived from human embryonic stem cells (hESCs) could effectively improve bladder urination function and repair its pathological features in animal models of interstitial cystitis/bladder pain syndrome (IC/BPS) induced by hydrochloric acid (Kim et al., 2017). The cell transplantation was beneficial for reconstructing urinary tract epithelium, reducing mast cell infiltration, alleviating tissue fibrosis, and preventing cell apoptosis. Within 12 months after implantation, there were no adverse reactions such as abnormal growth, tumor development, or immune transplant rejection. More importantly, the *in vivo* distribution and phenotypic characteristics of M-MSCs confirmed the long-term safety, graft survival rate, and *in vivo* characteristics of cell therapy for 6 months in live animal transplantation, providing reliable evidence for the treatment of hESC-derived MSCs for the first time. With the rapid development of stem cell research, stem cell therapy is expected to be successfully applied in clinical practice in urology in the near future. In addition, Song and colleagues transplanted human mesenchymal stem cells (hMSCs) overexpressing hepatocyte growth factor (HGF) into the inactive bladder wall caused by bladder outlet obstruction (BOO), significantly increasing the number of microvessels in the implantation area, reducing collagen deposition and cell apoptosis in the detrusor muscle, and restoring the contraction ability and motility of the rat bladder, as indicated by increased bladder contraction amplitude and reduced residual urine volume (Kim J. H. et al., 2021). This stem cell therapy strategy brings good news to patients with urinary system diseases and bladder dysfunction caused by BOO. Similarly, when ASCs were used to treat this type of diseases, significant bladder regeneration was achieved, including a decrease in TGF concentration, an increase in type I collagen, and a decrease in fibrosis degree (Siregar et al., 2022).

**FIGURE 4:**
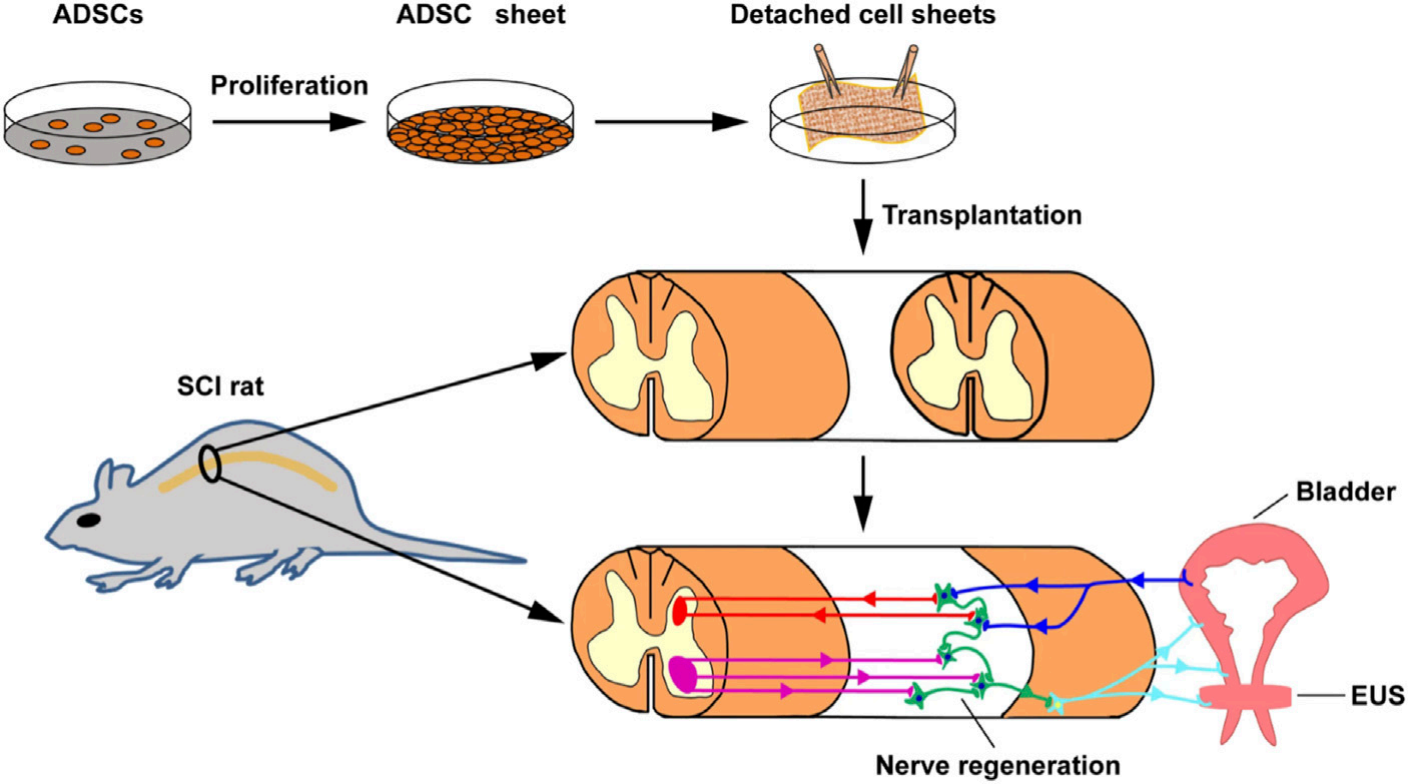
Schematic illustration of preparing and implanting an ADSC sheet into a rat SCI injury. Light blue line represents peripheral efferent nerve, deep blue line represent peripherals afferent nerve, red line represents supraspinal afferent pathway, and purple line represents supraspinal efferent pathway. Adapted from (Chen et al., 2022), Copyright 2022 Springer Nature.

To improve the delivery efficiency and therapeutic effect of stem cells, cell sheets have been developed and applied to enhance the efficacy of cell transplantation (Kim K. et al., 2021; Tang et al., 2023). In a study, Chen et al. prepared cell sheets of ASCs from adipose tissue of SD rats using temperature response method, and then transplanted the cell sheets into a spinal cord injury (SCI) rat model to investigate the function and pathological changes of the bladder (Figure 4) (Chen et al., 2022). Compared with the gelatin sponge implantation group, after 4 and 8 weeks of stem cell sheet transplantation, the bladder urination function caused by nerve injury in SCI rats was significantly improved, preventing damage to the bladder and urinary tract epithelium, thus maintaining the intact epithelial barrier, showing normal bladder wall morphology, reducing tissue fibrosis, and downregulating the expression of type 1 collagen. Undoubtedly, effective delivery of cell sheets will be a promising therapeutic option for bladder injury.

As a potential and effective cell source for cell therapy, *in situ* recruitment of endogenous stem cells at or around tissue injury sites has also received attention from researchers (Safina and Embree, 2022; Wei et al., 2023). For example, Jin et al. recruited endogenous stem cells using defocused low-energy shock wave (DLSW) and evaluated their specific effects on bladder repair (Jin et al., 2017). The urinary interval of the DLSW treatment group was significantly shortened, with a decrease in urine volume per urination and maximum urine pressure, indicating the recovery of bladder function after treatment. In addition, the DLSW treatment also significantly enhanced bladder nerve innervation, angiogenesis, and muscle regeneration, manifested as a significant improvement in damaged type IV collagen staining. Cell labeling and tracking results showed that the repair and functional improvement of bladder tissue originated from the recruitment of endogenous stem cells stimulated by DLSW, leading to the release of a large number of nerve growth factor (NGF) and VEGF. ASCs cultured *in vitro* also showed stronger migration ability, with higher expression level of stromal cell-derived factor-1 (SDF-1α) and higher secretion level of NGF and VEGF. This indicates that the biophysical therapy of DLSW can improve bladder repair and physiological function by recruiting endogenous stem cells, thereby enhancing bladder nerve innervation and angiogenesis during regeneration by secreting growth factors.

**FIGURE 5:**
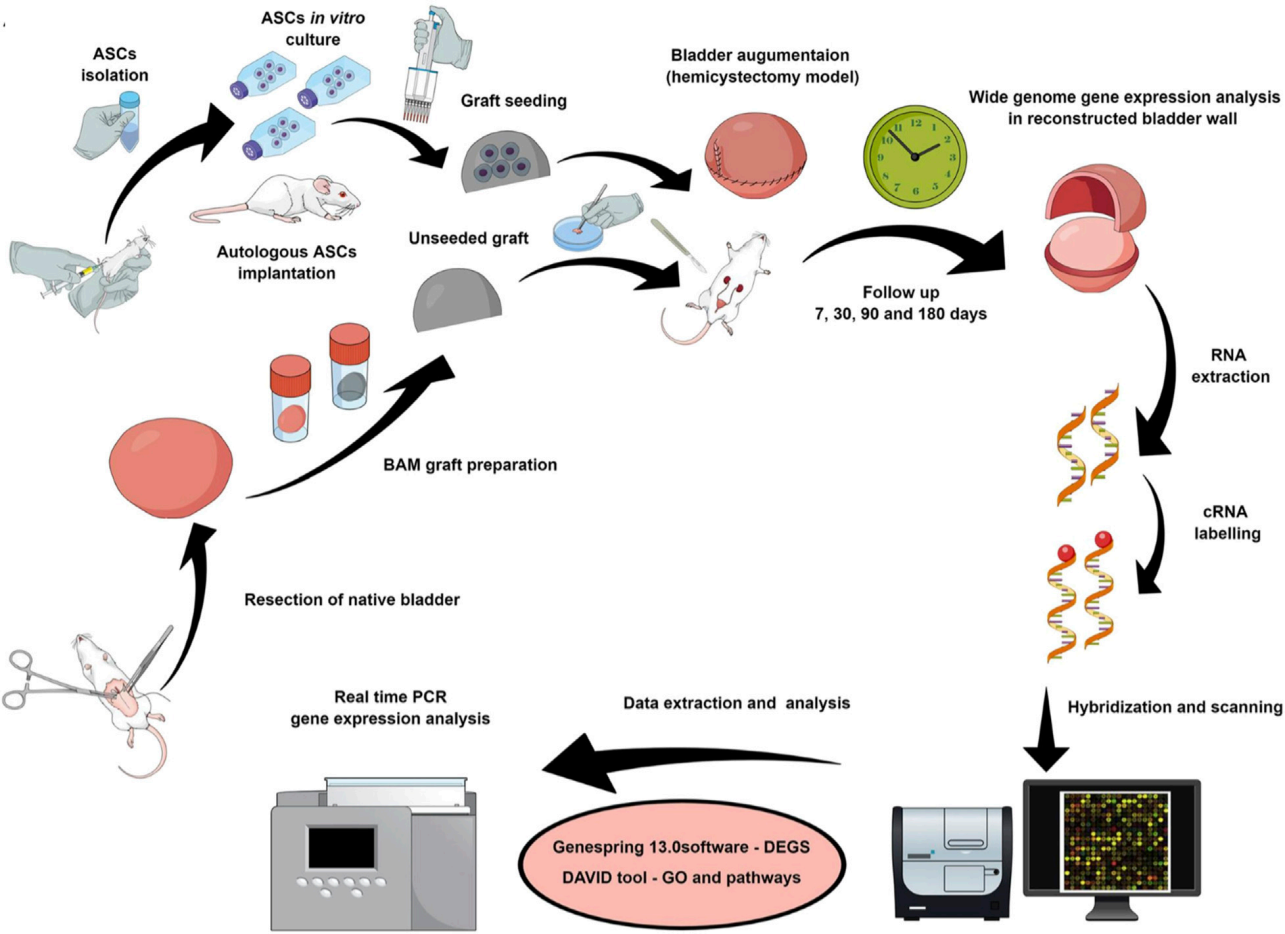
Schematic diagram of experimental design and process. Bladder was repaired using BAM scaffold with or without seeding ASCs. Adapted from (Pokrywczynska et al., 2019), Copyright 2019 Springer Nature.

The specific molecular mechanism of stem cell induced regeneration is crucial in the process of bladder regeneration. Therefore, Pokrywczynska et al. explored specific signaling pathways associated with bladder regeneration during the reconstruction of rat bladder using ASCs (Figure 5) (Pokrywczynska et al., 2019). They used BAM inoculated with ASCs to reconstruct damaged bladder. Macroscopic histological analysis and molecular techniques were used to analyze the potential molecular mechanisms of bladder healing within 180 days, and gene expression was analyzed using microarray, confirmed by real-time polymerase chain reaction (PCR). The authors found that using ASCs to repair bladder tissue could generate many differentially expressed genes, especially the Hedgehog signaling pathway, which includes genes such as Bmp2, Bmp4, Wnt2, Wnt2b, Wnt4, Wnt5a, Wnt10, etc. This study confirms that ASCs can alter the molecular pattern of bladder healing and trigger signaling pathways that may be associated with bladder regeneration, providing a reliable target for better induction of bladder regeneration in the future. Especially during bladder repair, ASCs can significantly upregulate the Hedgehog signaling pathway. Another similar study used similar materials for bladder repair in larger animal pigs (Pokrywczynska et al., 2018). The results showed that ASCs indeed promoted the regeneration of large defect bladder damage and confirmed the crucial role of stem cell paracrine effect in the repair process. Similar to the above reports, the combination of autologous endothelial progenitor cells (EPCs) with VEGF and PDGF-BB bioactive factors indicated that EPCs could directly participate in angiogenesis and have been proven to be an effective route for bladder regeneration by combining multiple pathways (Yang et al., 2024).

### 6.2 Organoids

Unlike stem cell-based cell therapy methods, organoids are 3D cultures that simulate *in vivo* organ characteristics using stem cells, providing new tools and platforms for disease mechanism research, diagnosis and treatment (Zhao et al., 2022). In the past few decades, organoids have been considered one of the most important scientific advances, revolutionizing research in multiple fields and becoming increasingly advanced and beneficial year by year. Organoids can perfectly solve the biggest challenge of studying body organs, which means there is almost no room for error (Vasyutin et al., 2019).

In a related study, Shin and colleagues used bladder stem cells to create multi-layered organoids that replicated structures extremely similar to natural tissues, with a stromal layer surrounded by urothelium and muscular layer (Kim E. et al., 2020). These organoids represented the cellular composition and gene expression level of the natural bladder at the single-cell level, maintaining the integrity of the urinary tract epithelium by forming tight junctions and plaques on the top surface of umbrella cells, and demonstrating the dynamic characteristics of *in vivo* regeneration through the proliferation of basal epithelial cells after injury. This organoid platform is very beneficial for a deeper understanding of the microenvironment of bladder tissue injury and further research on functional repair and regeneration. In another study, Suda et al. constructed bladder based organoids by incubating bladder urothelial cell suspensions in Matrigel and transplanted them onto a segment of mouse de-epithelialized colon to replace and create a hybrid urothelial-lined colon (Suda et al., 2023). It was worth noting that the outer layer of organoids cultured from adult mouse bladder contained typical Cytokeratin five positive (CK5+) cells, indicating that most basal organoid cells were in an undifferentiated state. After surgery, the bladder organoid cells formed multiple cell layers on the surface of colon lumen, reaching 4.58 ± 1.3 layers on the 28th day. Most cells highly expressing the proliferation marker protein Ki-67 (Ki67+) were located within these cell layers. This is the first report of successful implantation of bladder organoids in a de-epithelialized colon, completely reconstructing the urinary tract epithelial tissue composed of active proliferating cells.

From the discussion in this section, it can be seen that cell therapy and organoid technology have significant advantages and broad prospects in bladder regeneration. This is because transplanting normal or functionally specific cells or stem cells into damaged bladder to promote tissue regeneration and functional recovery has many advantages such as efficient, long-lasting treatment, broad adaptability, and relatively small side effects. However, cell therapy also faces some challenges, such as the need for a high quantity and high activity of cells, as well as significant investment and necessary equipment support. Organoid technology is based on specific culture of stem cells *in vitro*, highly simulating bladder tissue. It can provide more human like models to assist in the study of the physiological and pathological processes, regeneration and repair mechanisms, as well as drug screening and toxicity testing of bladder diseases. However, the cultivation of organoids requires significant investment, while also facing some ethical and legal issues. In short, both technologies face enormous development opportunities and policy bottlenecks, and the future is still full of hope.

## 7 Problems and prospects

Although the challenges faced by bladder regeneration still exist, it still demonstrates enormous potential and broad future prospects with the development of various innovative biomaterials and technologies summarized in this review (Table 2). Just as a scientist has pointed out, “Although we may be closer to identifying suitable biomaterials, we have not yet considered many factors that could lead to the failure of biomaterials as bladder substitutes (Farhat, 2011).” Currently, using biomaterials for bladder engineering or regeneration still seems too simplistic to truly find substantial breakthroughs in biomimetic structures and functions. Because the bladder tissue may look like a simple cystic organ, but in reality, it is not as simple as it appears and is not easy to regenerate and replace. It seems that we should learn more about the development, damage, and remodeling of the bladder to help understand how to better reconstruct its microstructure, mechanical properties and biological function *in vitro*.

**TABLE 2:** Biomaterials for promoting bladder repair.

Materials	Sources	Application approaches	Advantages	Limitations	References
Acellular ECM	BAM, goat bladder, dSIS, dUBM, dBP, dBD, dHD, colon, Lyoplant mesh, prepuce collagen, bovine skin collagen, autologous collagenous Biosheet	Implanting with ASCs, UCMSCs, long-chain aliphatic molecules, procyanidins, lipid nanospheres, CBD-bFGF	ASCs differentiating into mature SMCs, reducing fibrosis, regenerating bladder wall, improving compliance, alleviating pro-inflammatory of macrophages, anti-calcification, anti-inflammatory and antioxidant, regenerating urinary tract epithelium and muscle regeneration, neovascularization	Insufficient simulation of the microenvironment of chronic bladder inflammation such as poor blood supply and fibrosis, early immune responses in urodynamic environment	Pokrywczynska et al. (2022), Vishwakarma et al. (2020), Jin et al. (2020), Winde et al. (2019), Zhang et al. (2021), Sharma et al. (2022), Iimori et al. (2020)
Hydrogels	BAM, alginate dialdehyde and gelatin, gelatin and alginate, oxidized dextran/carboxymethyl chitosan, Pluronic F127	Loading PIECs, ASCs or exosomes, PCL nanofibers, BMHP and VP, PLGA nanoparticles, VEGF, bFGF	Revascularization, reducing fibrosis and inflammation, enhancing smooth muscle regeneration, nerve innervation, reducing graft contracture, regenerating urinary tract epithelial cells, promoting bladder repair	Insufficient observation of larger animals and long-term follow-up, impact of donor related variations on treatment differences	Liu et al. (2019), Xiao et al. (2021), Zhao et al. (2024)
Nano-biomaterials	SF, PLCL and PCL nanofibers, Graphene, ANS, ZIF-8 nanoparticles	Fe_3_O_4_NP, VEGF, ASC sheets, HA, ATE, hMDSCs, PIECs, Amniotic membrane, SMCs, UCs, EFS, SVF, PLGA microspheres	Accelerating angiogenesis, increasing conductivity, enhancing organized growth pattern of SMCs and UCs, M2 polarization of macrophages, bladder regeneration	The minimum safe dose of VEGF that can induce angiogenesis, requiring tests on larger animals, need to evaluate macrophage polarization effect	Wang et al. (2022c), Feng et al. (2019), Wang et al. (2022b), Adamowicz et al. (2020), Zhao et al. (2023)
Porous scaffolds	PRS, PEDOT, POCO, PCL scaffolds	bFGF, VEGF, EGF	Regenerating urinary tract epithelium and smooth muscle, promoting bladder regeneration	No clinically translatable animal model capable of replicating a human neurogenic bladder	Bury et al. (2024), Kim et al. (2020a), Am (2024)
Cell therapy	M-MSCs derived from hESCs, hMSCs overexpressing HGF, ASCs, DLSW	Transplantation, ASC cell sheets, recruiting endogenous stem cells, EPCs, BSMCs	Reconstructing urinary tract epithelium, alleviating tissue fibrosis, reducing collagen deposition, restoring bladder contraction ability and motility, improving nerve function, reducing urinary pressure, angiogenesis, muscle regeneration	Risk of tumorigenesis, lack of *in vivo* long-term analyses, insufficient efficiency and reliability for clinical application	Kim et al. (2021b), Tang et al. (2023), Pokrywczynska et al. (2019)
Organoids	Bladder stem cells, bladder urothelial cell suspensions	Multiple layers, matrigel	Forming tight junctions and plaques, re-epithelialization, forming multiple cell layers	Need large-scale animal research and long-term outcome evaluation	Kim et al. (2020b), Suda et al. (2023)

Abbreviations: BAM, bladder acellular matrix; dSIS, decellularized porcine small intestinal submucosa; dUBM, decellularized urinary bladder matrix; dBP, decellularized bovine pericardium; dBD, decellularized bovine dermis; dHD, decellularized human dermis; NPMyoD, nanoparticles containing myogenic differentiation 1 activated RNA; CBD-bFGF, collagen-binding domain-bFGF; PIECs, porcine iliac endothelial cells; ASCs, adipose stem cells; PCL, poly(ε-caprolactone); BMHP, bone marrow homing peptide; VP, vascular endothelial growth factor-mimicking peptide; PLGA, poly(lactide-co-gycolide); VEGF, vascular endothelial growth factor; bFGF, basic fibroblast growth factor; SMCs, smooth muscle cells; ECM, extracellular matrix; UCMSCs, human umbilical cord blood mesenchymal stem cells; SF, silk fibroin; Fe_3_O_4_NP, iron oxide nanoparticles; HA, hyaluronic acid; PLCL, poly(L-lactide-co-caprolactone); ATE, adipose tissue extract; hMDSCs, human muscle-derived stem cells; PRS, poly(1,8-octamethylene-citrate-co-octanol); BMSCs, bone marrow stem cells; EGF, epidermal growth factor; PEDOT, poly (3,4-ethylenedioxythiophene); POCO, poly (octamethylene-cooctanol citrate); UCs, urothelial cells; EFS, electric field stimulation; ANS, acellular nanocomposite scaffold; ZIF-8, zeolite imidazole salt skeleton-8; SVF, stromal vascular fraction, hESCs, human embryonic stem cells; M-MSCs, multipotent stem cells derived from hESCs; hMSCs, human mesenchymal stem cells; HGF, hepatocyte growth factors; DLSW, defocused low-energy shock wave; EPCs, endothelial progenitor cells; BSMCs, bladder smooth muscle cells.

The rapid development of tissue engineering, especially in biomaterials and stem cells, has indeed laid a solid foundation for bladder regeneration. At present, some medical products have been successfully applied in clinical practice, such as Lyoplant (collagen film, B. Braun, Germany), Cystatat (hyaluronic acid sodium solution, Mylan Institutional, Ireland), Ialuril (hyaluronic acid sodium solution, IBSA, Switzerland), Biodesign (SIS, Cook Medical Incorporated, USA), Symbotex (polypropylene mesh, Covidien, UK), etc. Although we have discussed the unique advantages of various materials, such as the composition and microstructure of acellular matrix closer to natural tissues, the high water content of hydrogels, the micro and nano structure of nanofibers, and so on. However, there are still obstacles in finding ideal biomaterials as bladder substitutes or auxiliary repair tools. The most common influencing factors are the pore structure and porosity of the materials, as they directly affect whether the material can be well cellular and vascularized. In addition, it is also closely related to permeability, as there may be a contradiction between permeability and porosity. Insufficient permeability may hinder cell transformation, affect bladder repair, and lead to fibrosis. Finally, as summarized and compared in Table 3, mechanical performance can also have a significant impact, limiting the ability of bladder repair, contraction, and other functions.

**TABLE 3:** Summary of mechanical properties of the biomaterials for bladder regeneration.

Materials	Preparation methods	Mechanical properties	References
BAM scaffold	Decellularization	76.30 ± 16.53 kPa (Young’s elastic modulus)	Pokrywczynska et al. (2022)
dBP	Decellularization	0.94 ± 0.16 MPa (Ultimate Tensile strength)	Sharma, et al. (2022)
dSIS	Decellularization	3.01 ± 0.32 MPa (Ultimate Tensile strength)	Sharma, et al. (2022)
dHD	Decellularization	4.50 ± 1.1 MPa (Ultimate Tensile strength)	Sharma, et al. (2022)
BAM hydrogel	Gelation	2.44 ± 0.41 kPa (Compressive modulus)	Liu et al. (2019)
BAMG-HS	Decellularization, knitting, gelation	5.33 ± 0.96 MPa (Elastic modulus)	Xiao et al. (2017)
PCL-ODex/CMC-PCL	Electrospinning, gelation	11.3 ± 0.7 N (Tensile load)	Zhao et al. (2024)
ATE/HA-PLCL	Electrospinning, lyophilization	14.93 ± 0.13 MPa (Tensile Young’s modulus)	Wang et al. (2022a)
PEDOT-POCO	Polymerization *in situ*	630 ± 188 kPa (Tensile Young’s modulus)	Ameer et al. (2024)

Abbreviations: BAMG-HS: BAM matrix graft-alginate dialdehyde-gelatin hydrogel-silk mesh; PCL-ODex/CMC-PCL, poly(ε-caprolactone) nanofibrous mats-oxidized dextran (ODex)/carboxymethyl chitosan hydrogel-poly(ε-caprolactone) nanofibrous mats; ATE/HA-PLCL, adipose tissue extract/hyaluronic acid-poly(L-lactide)/poly(ε-caprolactone).

The directional differentiation of stem cells into UCs or SMCs is also an important component of bladder regeneration, which determines whether the engineered or repaired bladder tissue has a complete multi-layer structure and sufficient mechanical properties. Stem cells inoculated onto scaffolds not only require specific vitality, but also appropriate growth factors or cytokines to regulate their growth and differentiation. Sometimes, physical and mechanical stimuli are also meaningful, as they together constitute the key inducing factors in the cellular differentiation microenvironment. Many physiological systems in the human body including heart valves, bladder, etc., involve biomechanics because mechanical stimulation occurs at multiple scales such as organs, tissues, cells, and produces biological effects (Sacks and Yoganathan, 2007). It is reported that the biomechanical stimulation has a significant impact on the differentiation of endothelial progenitor cells and mesenchymal stem cells into smooth muscle cells, among which physiological hemodynamic regulation has been found to promote the development of new tissues (Gandaglia et al., 2011; Suarez et al., 2021; Woodbury et al., 2023). Therefore, cell biology, biomaterials, and developmental biology must all play an appropriate role in the process of engineering bladder tissues *in vitro* or regenerating damaged bladder *in vivo*, achieving human compliance and functional contraction and urination in terms of structure, function, durability, and immune compatibility, which is truly beneficial for clinical practice.

Another promising direction for the future is from that the bladder has a certain self-healing ability. A study found that there were specialized cells in the inner layer of the bladder to repair tissue and restore the barrier against harmful substances concentrated in urine. Researchers found that non bladder cells in the Wolffian tube anatomy of mice could compensate for the loss of bladder cells, promote cell migration to the bladder, form bladder like features, and help restore organ function (Joseph et al., 2018). This surprising discovery is of great significance for the potential treatment of bladder injury and related diseases, as well as for improving the quality of life of patients related to bladder function and incontinence. In addition to this, there are some innovative technologies that will greatly help improve the treatment of bladder regeneration, such as using surgical robots to “reconstruct” a new bladder at the damaged bladder site. In this surgical procedure, a segment of intestinal tube is selected and de tubular to create a urine storage bladder that can store urine. Then, the ureters on both sides are implanted on the urine storage bladder, and then the urine storage bladder is anastomosed with the urethra. This allows patients to urinate through the original urethra as before removing the bladder. Patients do not need to wear urine collection bags, greatly improving their postoperative quality of life. Moreover, *in situ* implantable intelligent artificial bladder has also been preliminarily commercialized. It consists of a urine delivery and storage system and an intelligent automation system. After removing the damaged human bladder, the artificial bladder is implanted *in situ* into the patient’s pelvic cavity and anastomosed to reconstruct the urine delivery and storage system. It enables patients to independently control urination with minimal trauma. This mechanical structure device is expected to be widely used in clinical practice.

## 8 Conclusion

In this review, we first discussed the research progress of bladder regeneration based on innovative biomaterials such as acellular matrix, hydrogels, nanomaterials and porous scaffolds. Then, a brief introduction was given on the application and future development trends of emerging strategies such as cell therapy and organoids in bladder reconstruction. In addition, in response to these research advances, issues that need to be addressed and potential directions for future development have been proposed. It is believed that by addressing these problems and predicting directions, in the near future, more functional biomaterials and innovative technologies will greatly promote the development of bladder tissue engineering, helping researchers and clinical doctors realize its enormous potential advantages. After a certain stage of development, innovative biomaterials and related new technologies and platforms will undoubtedly provide better structure and performance for bladder regeneration and functional recovery than traditional autografts. This will be the ultimate goal of urology researchers and doctors, and may also provide patients with a better quality of life in the near future.
